# Effects of prebiotic oligofructose-enriched inulin on gut-derived uremic toxins and disease progression in rats with adenine-induced chronic kidney disease

**DOI:** 10.1371/journal.pone.0258145

**Published:** 2021-10-06

**Authors:** Ebru Melekoglu, M. Alper Cetinkaya, S. Evrim Kepekci-Tekkeli, Oguz Kul, Gulhan Samur

**Affiliations:** 1 Nutrition and Dietetics Department, Faculty of Health Sciences, Hacettepe University, Ankara, Turkey; 2 Laboratory Animals Application and Research Center, Hacettepe University, Ankara, Turkey; 3 Department of Analytical Chemistry, Faculty of Pharmacy, Bezmialem Vakıf University, Istanbul, Turkey; 4 Department of Pathology, Faculty of Veterinary Medicine, Kirikkale University, Kirikkale, Turkey; INRAE, FRANCE

## Abstract

Recent studies suggest that dysbiosis in chronic kidney disease (CKD) increases gut-derived uremic toxins (GDUT) generation, leads to systemic inflammation, reactive oxygen species generation, and poor prognosis. This study aimed to investigate the effect of oligofructose-enriched inulin supplementation on GDUT levels, inflammatory and antioxidant parameters, renal damage, and intestinal barrier function in adenine-induced CKD rats. Male Sprague-Dawley rats were divided into control group (CTL, n = 12) fed with standard diet; and CKD group (n = 16) given adenine (200 mg/kg/day) by oral gavage for 3-weeks to induce CKD. At the 4th week, CKD rats were subdivided into prebiotic supplementation (5g/kg/day) for four consecutive weeks (CKD-Pre, n = 8). Also, the control group was subdivided into two subgroups; prebiotic supplemented (CTL-Pre, n = 6) and non-supplemented group (CTL, n = 6). Results showed that prebiotic oligofructose-enriched inulin supplementation did not significantly reduce serum indoxyl sulfate (IS) but did significantly reduce serum p-Cresyl sulfate (PCS) (p = 0.002) in CKD rats. Prebiotic supplementation also reduced serum urea (p = 0.008) and interleukin (IL)-6 levels (p = 0.001), ameliorated renal injury, and enhanced antioxidant enzyme activity of glutathione peroxidase (GPx) (p = 0.002) and superoxide dismutase (SOD) (p = 0.001) in renal tissues of CKD rats. No significant changes were observed in colonic epithelial tight junction proteins claudin-1 and occludin in the CKD-Pre group. In adenine-induced CKD rats, oligofructose-enriched inulin supplementation resulted in a reduction in serum urea and PCS levels, enhancement of the antioxidant activity in the renal tissues, and retardation of the disease progression.

## Introduction

Chronic kidney disease (CKD) is characterized by progressive glomerular, tubular, and interstitial damage, in which case metabolic end products, called uremic toxins, accumulate in the body [1]. One of the most important sources of uremic toxins is gut microbial metabolism, furthermore, a complex two-sided relationship exists between gut and CKD. The uremic state in CKD causes increased generation of gut-derived uremic toxins (GDUT) such as indoxyl sulfate (IS) and p-Cresyl sulfate (PCS) as a consequence of changes in gut microbiota composition and metabolism [2]. Due to renal function decreases in CKD, uremic toxins cannot be effectively removed from the body and are accumulated in serum and body tissues, thus contributing to CKD progression, immune dysregulation, and uremic symptoms that adversely affect the cardiovascular, endocrine, and neurological systems [3].

Gut dysbiosis in patients with CKD has multifactorial causes including uremic state, metabolic acidosis, pharmacological therapies (antibiotics, phosphate binders), slow colonic transit, and dietary restriction of potassium-rich fruits and vegetables [4, 5]. Dietary restriction of fruits and vegetables, a major source of fermentable dietary fibers, hinders the transformation of dietary fibers to short-chain fatty acids (SCFA), which are the primary nutrient for the symbiotic gut microbiota [5]. Lack of fermentable dietary fiber in the colon and a slowing down in colonic transit time induce bacterial protein fermentation and formation of GDUT [6]. Low dietary fiber intake can also increase intestinal permeability, transportation of endotoxins via the circulatory system, and chronic inflammation and aggravate CKD progression [2, 7].

Therapeutic interventions such as the use of prebiotics, probiotics, or symbiotics for lowering the gut synthesis of IS and PCS have emerged as a potential strategy to modify the adverse effects induced by gut dysbiosis in CKD [8–12]. Prebiotics may be linked to the shift of gut microbial metabolism from a proteolytic fermentation to a saccharolytic fermentation pattern. Accordingly, GDUT formation may also be reduced in CKD. Growing evidence has highlighted that dietary fermentable fiber exhibited positive effects on inflammation, oxidative stress, integrity and function of the gut barrier, and renal injury in experimental models with CKD [9, 11, 13].

Inulin is a fermentable fiber that is naturally found in wheat, onion, banana, artichoke, however, the commercially available form is mostly derived from chicory root [14]. The most active product among the inulin-type fructans is oligofructose-enriched inulin [15]. Meijers et al. [16] demonstrated that oligofructose-enriched inulin reduces serum p-cresol levels in hemodialysis patients. However, limited data is available regarding the effect of prebiotic oligofructose-enriched inulin on inflammation, oxidative stress, gut barrier function, and renal injury in CKD. Thus, this study aimed to investigate the therapeutic potential of oligofructose-enriched inulin administration on GDUT levels, inflammatory and antioxidant parameters, renal damage, and intestinal permeability in adenine-induced CKD rats.

## Materials and methods

The study was conducted at the Hacettepe University Laboratory Animals Application and Research Centre. All experimental protocols were approved by the Animal Experimentations Local Ethics Board of the Hacettepe University, Turkey (ethical committee approval number: 2017/37-07).

### Animals

Male Sprague-Dawley rats (n = 28) weighing 350 ± 20 g were obtained from Kobay Inc. (Ankara, Turkey) and housed in separate cages under a 12-h light/dark cycle at ~22°C and 55–60% relative humidity. Rats were fed a standard chow diet (5% fat, 23% protein (w/w), energy 2.6 kcal/g, Bil-Yem Ltd. Co., Ankara, Turkey). All rats had free access to chow and water ad libitum and their daily food and water intake were recorded. The rats were observed twice daily for any signs of adverse events or suffering. We made all efforts to minimize the animals’ suffering, all invasive procedures were performed under isoflurane anesthesia.

### Chemicals

Adenine was obtained from Sigma-Aldrich Co. (St Louis, MO, USA) and was prepared freshly every day. Rat interleukin (IL)-6 (cat. no: CSB-E04640r) and IL-10 (cat. no: CSB-E04595r) enzyme-linked immunosorbent assay (ELISA) kits were purchased from Cusabio Technology LLC (Wuhan, Hubei, China). All immunohistochemical tests were performed using a commercial streptavidin-biotin kit (Thermo Fisher Scientific, USA) and aminoethyl carbazole (AEC) chromogen (Thermo Fisher Scientific, USA). Antibodies against superoxide dismutase (SOD) (sc-101523), glutathione peroxidase (GPx) (sc-133160), claudin-1 (sc-166338), and occludin (sc-133256) were purchased from Santa Cruz Biotechnology (Dallas, TX, USA).

### The adenine-induced rat model

After an acclimatization period of seven days, rats were randomized to the CKD (n = 16) and control groups (n = 12). The CKD rats were given 200 mg/kg body weight of adenine by oral gavage once a day for 3 weeks. Control animals were given only 1 ml drinking water once a day for 3 weeks by oral gavage instead of the adenine solution.

### Diet intervention

After an adenine administration for 3 weeks, the rats were subdivided into four groups as follows: (i) CKD rats with normal diet (CKD) (n = 8); (ii) CKD rats with prebiotic diet (CKD-Pre) (n = 8); (iii) control rats with normal diet (CTL) (n = 6); (iv) control rats with prebiotic diet (CTL-Pre) (n = 6). For prebiotic treatment, oligofructose-enriched inulin which is a commercialized food ingredient made of a mixture of long-chain inulin and oligofructose, Beneo Synergy1 was obtained from Orafti^®^ (Tienen, Belgium). Rats in the CKD-Pre and CTL-Pre groups were administered oligofructose-enriched inulin in their drinking water at a daily dose of 5 g/kg body weight for 4 weeks, whereas the CKD and CTL groups received no addition. The dose and administration duration of prebiotic were determined based on previous studies conducted in rats, which demonstrated positive effects on inflammation, oxidative stress, and gut microbiota modulation [8, 9, 17].

### Sample collection and biological examination

Body weight was measured every week. After 4 weeks of prebiotic supplementation, rats were euthanized under isoflurane anesthesia by cardiac puncture to obtain blood, kidney, and colon tissues. Concentrations of serum urea and creatinine were measured by standard analytical methods. Inflammatory markers, IL-6 and IL-10 were measured in the serum samples using commercially available ELISA kits according to the manufacturer’s instructions. The serum concentration of free IS and PCS were measured using high-performance liquid chromatography (HPLC; LC-10 AD, Shimadzu Corporation, Kyoto, Japan). 1 ml of serum samples of each group were separated from other serum constituents and determined by using a C18 ODS column with the dimensions; 4.6 mm I.D, 150 mm length and 5 μm particle size at 40°C (GL Sciences Inc. Tokyo, Japan). The mobile phase was acetonitrile-sodium acetate buffer at pH 4.5 (55:45 v:v) with a flow rate of 1 ml/min. Before injection to HPLC, 750 μl acetonitrile was added to each sample and mixed with a vortex mixer for 15 minutes and centrifuged for 30 seconds at 4000 rpm to provide serum protein precipitation. After this pre-treatment procedure, 20 μl of the supernatant of each sample was taken and injected into the HPLC system. HPLC was combined with fluorometric detection and IS and PCS were measured at 280 nm excitation and 330 nm emission wavelength.

### Histopathological examinations

A total of 56 kidneys (right and left) and 28 colon tissue samples were collected and fixed in 10% buffered formalin for 48–72 hours, following routine tissue processing procedures they were embedded in paraffin. Colon and kidney tissues were sectioned with a thickness of 5μm and routinely stained with Hematoxylin and eosin (H&E). Renal histopathologic damage, including crystalline deposition, tubular damage, glomerular damage, glomerular inflammation, and interstitial fibrosis was evaluated [18]. A semi-quantitative grading method was applied, details are as follows: The severity of the regarding pathological changes was evaluated in 5 different 20x objective fields, and degree of nephropathy was scored from 0 to 4 according to an area of coverage, (grade 0: no injury, grade 1: <25%, grade 2 = 25–50%, grade 3: 50–75%, grade 4: >75%), then calculated average [19].

### Immunohistochemical analysis

Avidin-biotin complex immunoperoxidase test was performed to demonstrate SOD, GPx, occluding, and claudin commercial antibodies were used on 5μm paraffin sections using the streptavidin-biotin-peroxidase complex (ABC) technique. All steps were carried out using the commercial kit’s protocol (Thermo Fisher Scientific, USA). Briefly, after deparaffinization of the sections, antigen retrieval was applied by boiling in citrate buffer (pH 6.0) for 20 minutes. Endogenous peroxidase activity was quenched with hydrogen peroxide 3% in absolute methanol for 7 minutes. Non-specific binding was blocked with a blocking serum for 5 minutes. Thereafter, tissue sections were then incubated with the following primary antibodies; SOD, GPx, claudin-1, and occludin for 60 minutes. After treating the sections with biotin marked secondary antibody for 15 minutes and with the streptavidin-peroxidase enzyme gain for 15 minutes at room temperature. Finally, sections were incubated in AEC chromogen for 5–10 minutes and Mayer’s hematoxylin was applied for counterstaining for 1–2 minutes and mounted with an aqueous mounting medium. As a negative control, the primary antibody step was omitted. Sections were analyzed using a trinocular microscope (Olympus BX51, Tokyo, Japan) and their microphotographs were taken.

### Quantitative histomorphometric analysis

Quantitative analysis of the percentage of positive staining regions SOD and GPx were calculated using The Olympus DP2-BSW (Ver.2.0, Tokyo, Japan) software system. This system consists of a camera (Olympus DP25) attached to a trinocular light microscope (Olympus BX51, Tokyo, Japan) and a computer with a software system. Briefly, immunopositively stained areas for each parameter (SOD and GPx) were automatically selected in three different 20x objective magnification views, and their area of percentage values was determined.

### Statistical analysis

The sample size was determined by power analysis (α = 0.05 and β = 0.20), based on the preliminary results of a pilot study. All statistical analyses were performed using Statistical Package for the Social Sciences (SPSS) software (IBM SPSS Statistics for Windows, Version 23.0. Armonk, NY). Data were first analyzed using Bartlett’s test for equality of variances. Statistical analyses were performed by one-way analysis of variance (ANOVA) followed by Student’s t-tests. Significance values were adjusted for multiple comparisons according to Bonferroni. All of the values are expressed as the mean ± SEM. A p-value of less than 0.05 was considered to show a statistically significant result.

## Results

### General data

Data are summarized in Table 1. Rats in both CKD groups weighed significantly less than the healthy control groups. There was no significant difference in body weight between the CKD and CKD-Pre (p = 0.06), and between CTL and CTL-Pre groups (p = 0.238). Daily food consumption (g/day) in the CKD group were significantly less than those in the CTL rats (p = 0.003). Serum urea and creatinine levels were used as indirect measures of glomerular filtration rate. Serum urea and creatinine concentration were significantly higher in the CKD rats compared with the CTL group. Serum urea concentration in the CKD-Pre group was significantly lower than those in the CKD group (p = 0.008) but still higher than those in both control groups. Serum creatinine concentration in the CKD-Pre group was not significantly different when compared with the CKD group.

**Table 1:** Physiological and biochemical parameters.

	CTL	CTL-Pre	CKD	CKD-Pre	p
**BW (g)**	505.2 ± 21.47^a^	476.8 ± 6.98^a^	375.4 ± 18.60^b^	421.3 ± 12.20^b^	<0.001
**Food intake (g/day)**	33.0 ± 0.97^a^	30.3 ± 1.17^a,b^	25.5 ± 1.66^b^	27.9 ± 0.89^b^	0.003
**Serum urea (mg/dl)**	41.9 ± 1.81^a^	39.0 ± 2.02^a^	127.4 ± 16.18^b^	73.2 ± 6.51^c^	<0.001
**Serum creatinine (mg/dl)**	0.6 ± 0.09^a,b^	0.5 ± 0.01^a^	1.3 ± 0.17^c^	0.8 ± 0.06^b,c^	<0.001
**Serum PCS (ng/ml)**	901.6 ± 114.95^a^	351.2 ± 48.47^b^	1771.1 ± 269.67^c^	571.7 ± 115.64^a,b^	<0.001
**Serum IS (μg/ml)**	94.8 ± 3.28^a^	89.2 ± 1.82^a^	134.1 ± 10.04^b^	102.2 ± 4.28^a,b^	<0.001
**Serum IL-6 (pg/ml)**	46.4 ± 14.75^a^	59.3 ± 30.42^a^	205.8 ± 27.17^b^	63.0 ± 16.57^a^	<0.001
**Serum IL-10 (pg/ml)**	59.2 ± 12.64	43.6 ± 9.69	103.8 ± 28.35	56.7 ± 11.38	0.135

Physiological and biochemical parameters of CTL, CTL-Pre, CKD, and CKD-Pre obtained at euthanasia day.

Data are presented as mean ± SEM.

Values not sharing common superscripts (^a, b, c^) are significantly different in a row using post hoc procedure.

CTL, control; CTL-Pre, control group fed oligofructose-enriched inulin; CKD, adenine induced chronic kidney disease group; CKD-Pre, CKD group fed oligofructose-enriched inulin; BW, body weight.

In our present study, the CKD group showed significantly higher serum IS and PCS concentration than healthy control rats. Although serum PCS concentration was significantly lower in the CKD-Pre group compared with CKD rats (p = 0.002), serum IS concentrations were not significantly different between both CKD groups. Also, serum PCS concentration in the CTL-Pre group was significantly lower than those in the CTL group (p = 0.003).

The CKD group showed significantly higher serum IL-6 concentration than healthy control rats. Serum IL-6 concentration in the CKD-Pre group was significantly lower than those in the CKD group (p = 0.001). Serum IL-10 concentration did not differ between the four groups (p = 0.135) (Table 1).

### Histopathological findings

**Fig 1:**
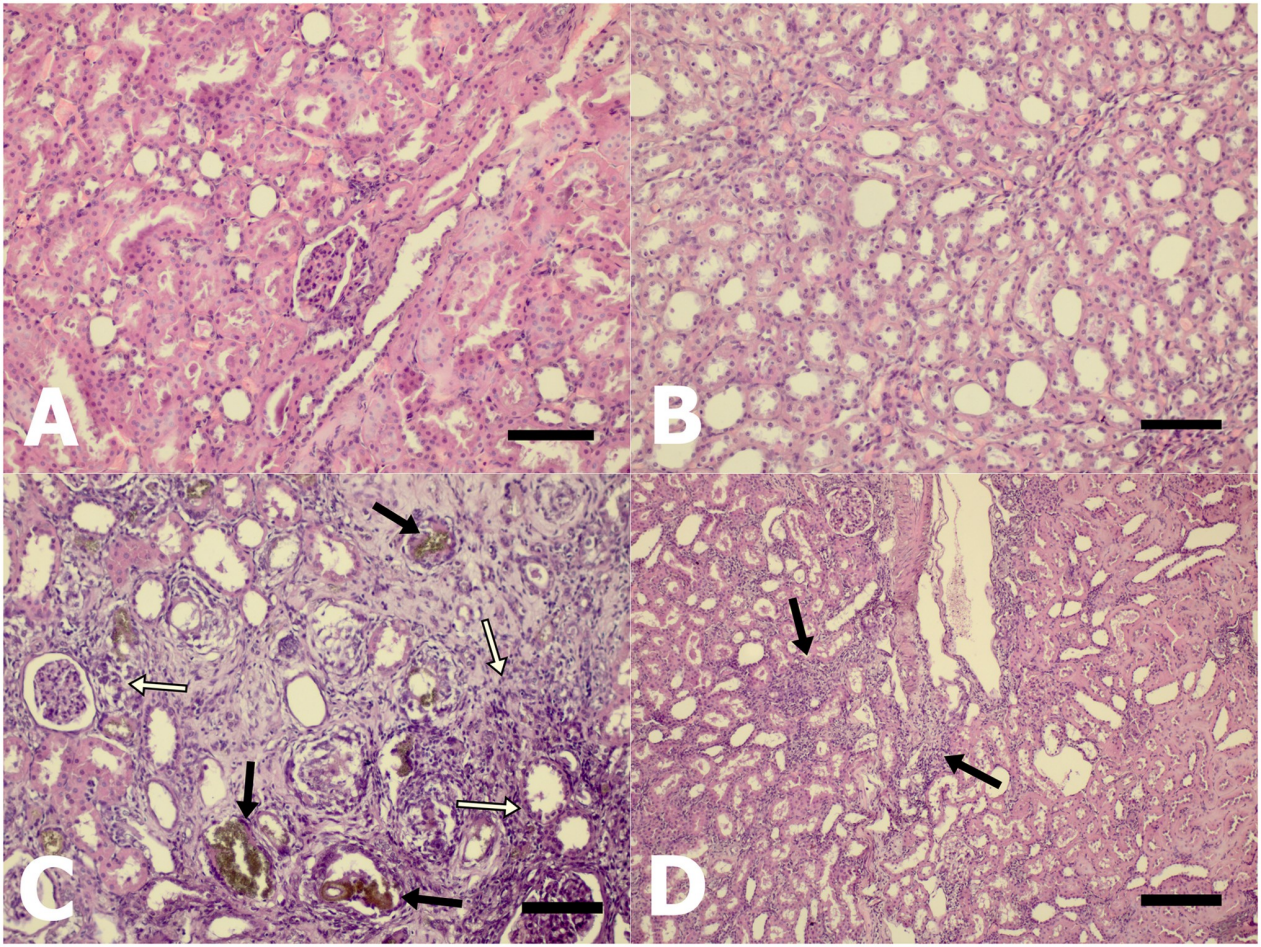
Histological kidney sections stained with H&E from (A) CTL, (B) CTL-Pre, (C) CKD, and (D) CKD-Pre. Scale bars: (A, B, C, D); 220 μm.

The kidneys of CKD rats showed cystic dilatation, degeneration, atrophy, and calcification of tubules, interstitial fibrosis, glomerulosclerosis, thickening of the Bowman’s capsule, glomerular atrophy, and the existence of giant cells associated with greater deposition of adenine crystals (Fig 1).

In the CKD-Pre group, multifocal areas of mild fibrosis were observed within the renal cortex. Reparative macrophages and polymorphonuclear leukocytes were increased in the CKD-Pre group compared with the CKD group. The crystalline deposition was very scarce or did not exist (Fig 1).

Histopathological findings were assessed semi-quantitatively, and the mean severity scores of the groups are presented in Table 2. Severity scores of crystalline deposition, tubular and glomerular damage, glomerular inflammation, and interstitial fibrosis were higher among CKD rats compared to the CKD-Pre group. Crystalline deposition, glomerular inflammation, and interstitial fibrosis were not observed in the kidneys of both control groups. Total severity scores of the CKD-Pre group were significantly lower than those CKD rats (p = 0.001). There was no significant difference in total severity scores between the CTL and CTL-Pre groups (p = 0.258).

**Table 2:** Renal histopathological findings.

	Semiquantitative mean severity score				
	CTL	CTL-Pre	CKD	CKD-Pre	
Crystalline deposition	0	0	3.1	0.9	
Tubular damage	0.6	0.4	3.6	1.8	
Glomerular damage	0.4	0	3.8	1.4	
Glomerular inflammation	0	0	3.6	2.2	
Interstitial fibrosis	0	0	3.8	2.0	
**Total injury score**	1.0 ± 0.37^**a**^	0.7 ± 0.21^**a**^	17.9 ± 0.95^**b**^	8.8 ± 1.19^**c**^	p<0.001

Total severity scores of the groups are presented as mean ± SEM.

Values not sharing common superscripts (^a, b, c^) are significantly different in a row using post hoc procedure.

A semi-quantitative grading method was applied, details are as follows: The degree of nephropathy changes was scored from 0 to 4, (grade 0: no injury, grade 1: <25%, grade 2 = 25–50%, grade 3: 50–75%, grade 4: >75%.

CTL, control; CTL-Pre, control group fed oligofructose-enriched inulin; CKD, adenine induced chronic kidney disease group; CKD-Pre, CKD group fed oligofructose-enriched inulin.

### Antioxidant enzyme activity in kidney tissues

**Fig 2:**
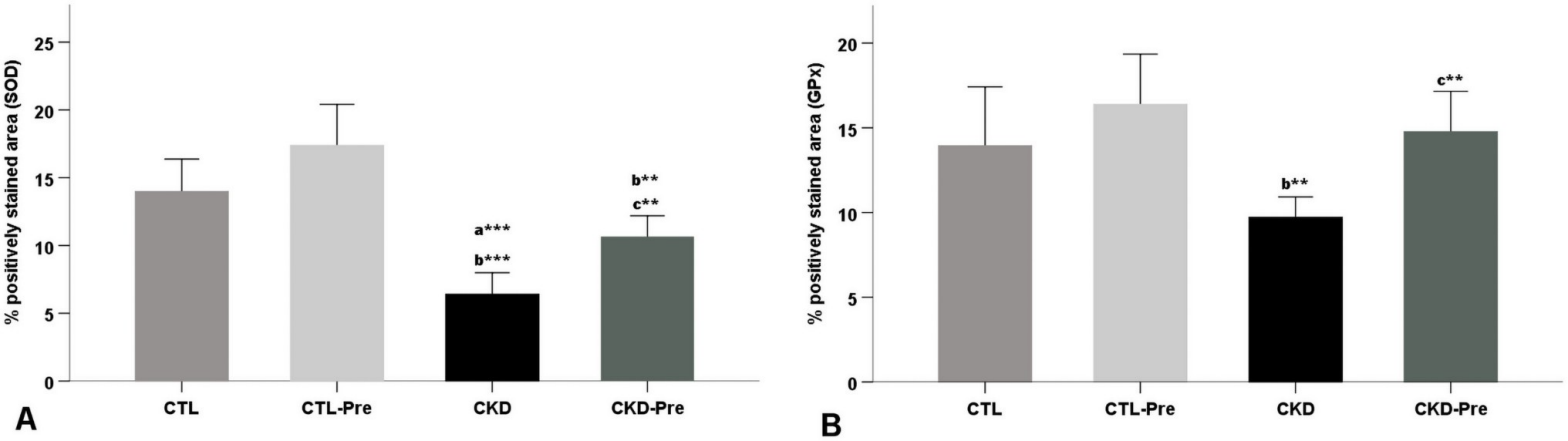
Bars represent the quantitative analysis of (A) SOD positively stained area (%) and (B) GPx positively stained area (%). Data are expressed as mean ± SEM. “a” significant difference compared to CTL group; “b” significant difference compared to CTL-Pre group; “c” significant difference compared to CKD group. *p<0.05; **p<0.01; ***p<0.001.

**Fig 3:**
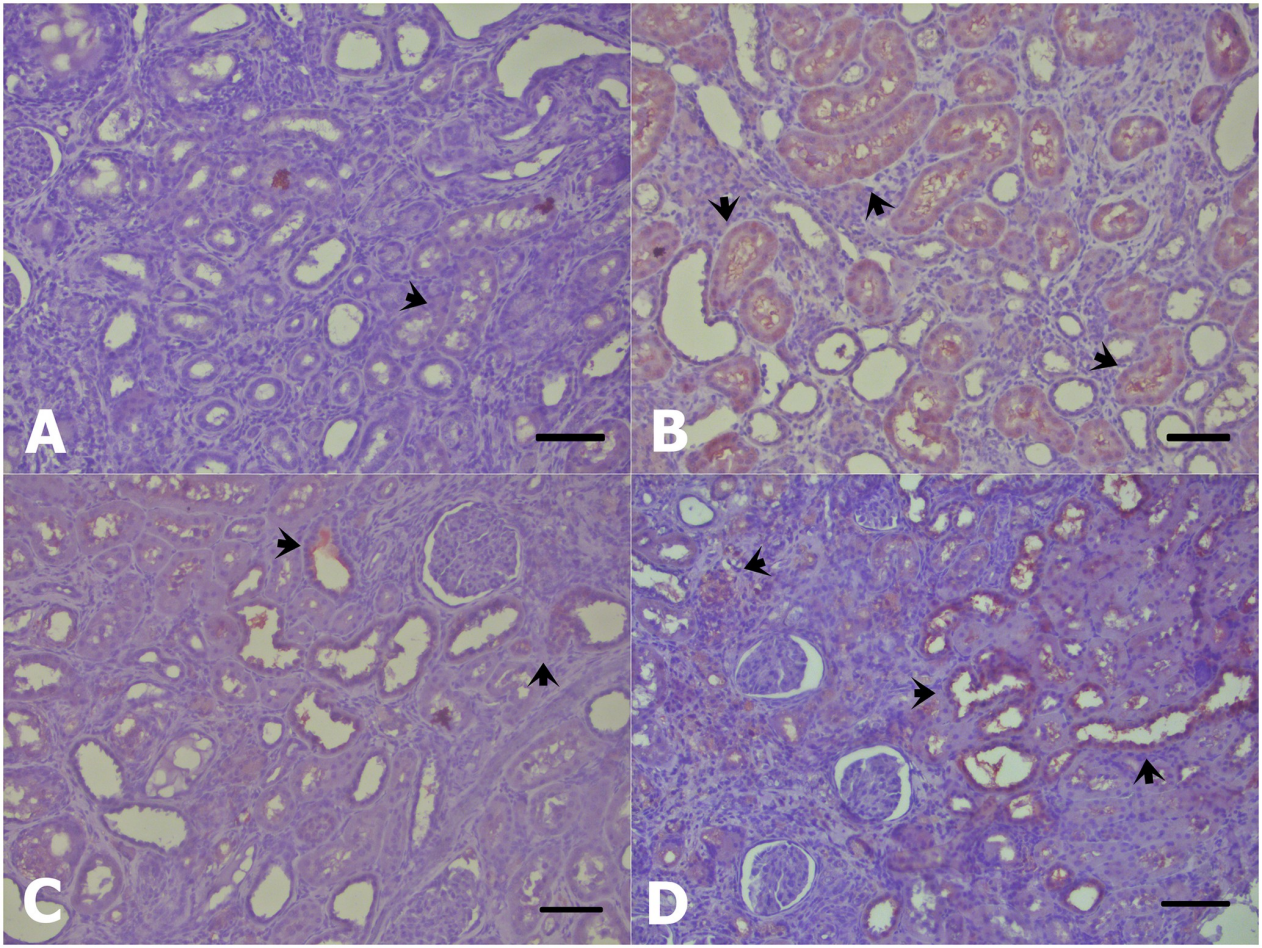
Immunopositive staining of SOD; (A) CKD, (B) CKD-Pre and GPx; (C) CKD, (D) CKD-Pre activities (arrowheads) in adenine-induced CKD. Scale bars: (A, B, C, D); 220 μm.

SOD and GPx activities for the kidney tissues are shown in Figs 2 and 3. Renal SOD and GPx activities increased significantly in the CKD-Pre rats versus in the CKD group (p = 0.001, p = 0.002, respectively). However, there was no significant difference between the CTL and CTL-Pre groups. Renal SOD activity decreased significantly in the CKD group when compared to the CTL group (p<0.001). Renal GPx activity was significantly lower in the CKD group in comparison to the CTL-Pre group (p = 0.001).

### Tight junction protein expression in colonic tissues

**Fig 4:**
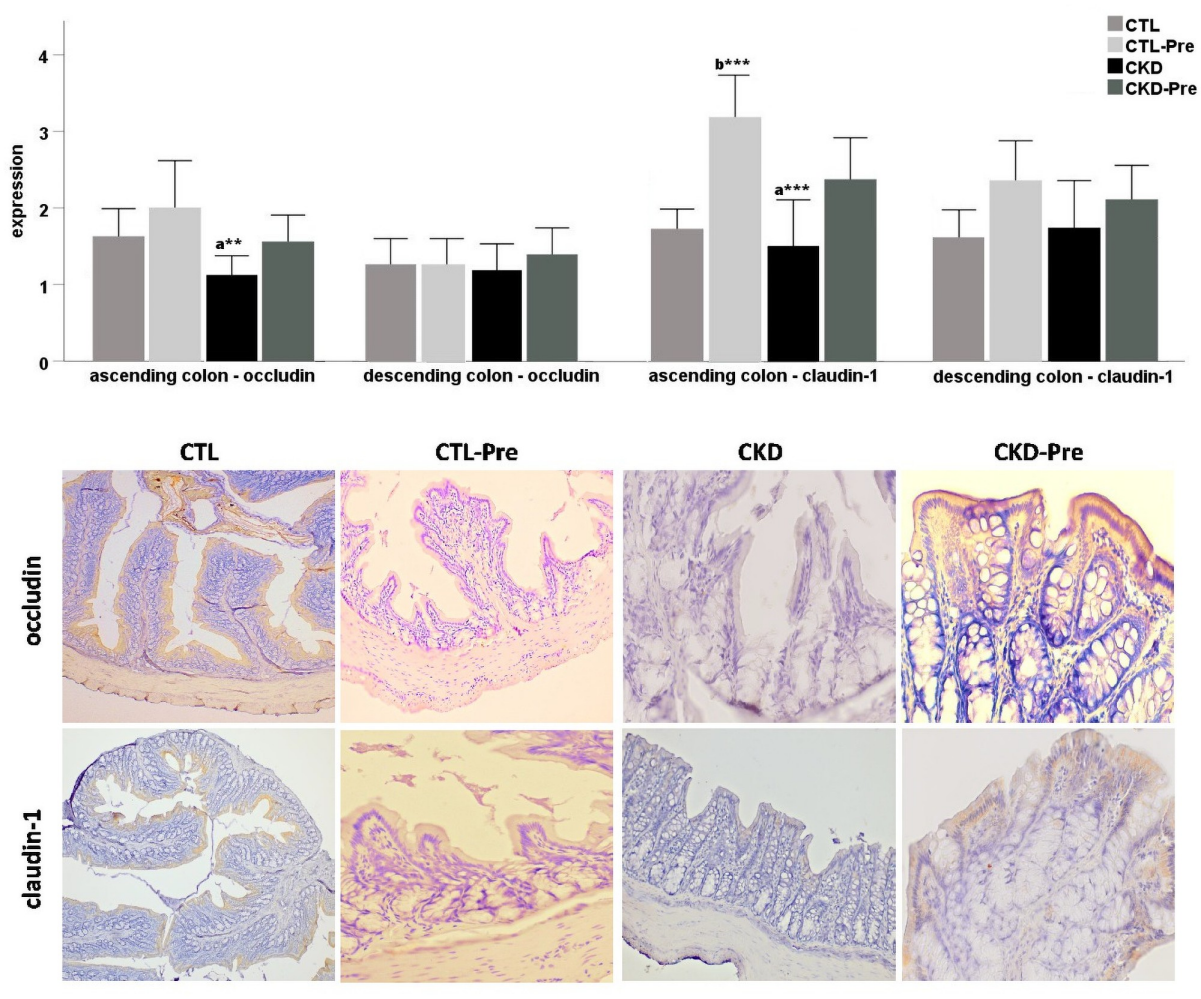
Immunoperoxidase expression of occludin and claudin-1 in the ascending and descending colon of CTL, CTL-Pre, CKD, and CKD-Pre groups. Indirect ABC immunoperoxidase technique, Mayer’s hematoxylin counterstaining. Immunohistochemical staining in all groups was scored semi-quantitatively from 0 to 4. Data are expressed as mean ± SEM. “a” significant difference compared to CTL-Pre group; “b” significant difference compared to CTL group. *p<0.05; **p<0.01; ***p<0.001. Representative images showing immunohistochemical staining for occludin and claudin-1 in ascending colon of CTL, CTL-Pre, CKD, and CKD-Pre. Scale bar = occludin: CTL, CTL-Pre 180 μm, CKD, CKD-Pre 90 μm; claudin-1: CTL 220 μm, CTL-Pre, CKD-Pre, 160 μm, CKD 280 μm.

Data are shown in Fig 4. Expression of occludin and claudin-1 was assessed in the ascending and descending colon of all groups. Occludin expression in the ascending colon was significantly increased in the CTL-Pre group when compared to the CKD group (p<0.01). Occludin expression in the ascending and descending colon was similar in the CKD group and the CKD-Pre group. Similarly, there was no significant difference in claudin-1 expression in the ascending and descending colon when CKD and CKD-Pre groups were compared. The CTL-Pre group showed a significant increase of claudin-1 expression in the ascending colon compared with the CKD and CTL groups (p<0.001).

## Discussion

As kidney function becomes less effective, uremic toxins accumulate in the body, with those are thought to be responsible for inflammation, gut dysbiosis, bacterial translocation, and CKD progression [20]. Herein, the adenine-induced CKD model was used to investigate the efficacy of the oligofructose-enriched inulin diet on GDUT formation, inflammation, oxidative stress, intestinal barrier permeability, and disease progression. Results confirmed the irreversible renal damage of adenine since its administration for 3 weeks significantly increased serum urea and creatinine level. Also, histopathological findings supported the irreversible renal damage in CKD rats. In the present study, we found that oligofructose-enriched inulin administration in CKD rats significantly decreased the concentrations of serum urea, PCS, and IL-6 and ameliorated renal injury.

The serum levels of IS and PCS indicate the balance between the generation of these GDUT and their elimination by the kidney. Renal clearance of protein-bound uremic toxins mainly depends on tubular secretion mechanisms [21, 22]. IS and PCS are excreted by renal tubular secretion and their clearance rates significantly higher than the glomerular filtration rate [23]. With CKD progression, renal clearance of IS and PCS decreases, and their plasma levels increase [23]. In the present study, serum levels of IS and PCS were significantly higher in the adenine-induced CKD rats compared with their healthy control group.

IS and PCS are derived from the gut microbiota metabolism of tryptophan and tyrosine. Intestinal fermentation of tryptophan by tryptophanase-containing bacterial species (*Lactobacillus*, *Bifidobacterium longum*, *Bacteroides fragilis*, *Parabacteroides distasonis*, *Clostridium bartlettii*, *Eubacterium hallii*) generates indoles and IS [24]. Intestinal fermentation of tyrosine by intestinal p-cresol-producing microbial species (*Clostridium difficile*, *Faecalibacterium prausnitzii*, *Bifidobacterium*, *Subdoligranulum*, *Lactobacillus*) generates p-cresol [7]. The interconnection between gut microbiota and kidney function, known as the gut–kidney axis, is a complex and two-sided relationship. On the one hand, the uremic state in CKD affects gut microbiota composition, on the other hand, gut microbiota composition causes the increase of GDUT production that might lead to CKD progression [2]. Given the promising effects of dietary fermentable fibers on modulating metabolism and composition of the gut microbiota, prebiotic administration may reduce serum GDUT levels. Previously, it has been shown higher rates of colonic fermentation of aromatic amino acids (tryptophan and tyrosine) in the setting of higher luminal pH [25, 26]. Furthermore, decreased phenolic and indolic metabolites production has been reported in the presence of fermentable dietary fiber and low luminal pH [25, 26]. Fermentation of dietary fiber by the gut microbiota results in SCFA production [27]. Increased production of SCFA decreases luminal pH, which consequently decreases GDUT formation [9]. In the present study, we found that in both CKD and healthy rats, oligofructose-enriched inulin administration decreased the serum PCS level significantly (Table 1). The reduction in the serum PCS levels might be due to changing the intestinal flora with oligofructose-enriched inulin and decreased gut microbiota generation of PCS [28]. We also found that oligofructose-enriched inulin administration has no significant effect on serum IS levels in CKD rats (Table 1). Our findings are consistent with a previous study by Yang et al., showing that supplementation of xylooligosaccharide decreased serum PCS, but not IS levels in adenine-induced CKD mice [29]. They analyzed microbiota composition using a computational method and reported that tyrosine metabolism was decreased with xylooligosaccharide supplementation [29]. However, there was no correlation between the change of tryptophan metabolism and serum IS levels [29].

In the present study, we examined free serum concentrations of the IS and PCS to which tissues throughout the body are exposed [30]. Kieffer et al. [8] examined the effects of resistant starch on uremic toxins in the serum, urine, and cecal contents and reported that resistant starch reduced serum and urine IS levels by 36% and 66% respectively, and urine PCS levels by 47% in rats with renal failure. Inconsistent with our findings, Furuse et al. [11] reported that galactooligosaccharides administration significantly reduced serum IS levels and modified the gut microbiota in the 5/6 nephrectomized rats. Sueyoshi et al. [31] demonstrated that lactulose supplementation significantly reduced serum IS and PCS levels in adenine-induced CKD rats. One possible reason for these inconsistencies might be different types of prebiotics used to modulate gut microbiota [32]. Recently, it has been pointed out that the use of certain prebiotic and probiotic strains might reduce the generation of certain GDUT [33]. Considering our results, we speculated that oligofructose-enriched inulin had no significant effect on the gut microbial metabolism of tryptophan. Devlin et al. [34] colonized the gnotobiotic mice with tryptophanase-containing *Bacteroides thetaiotaomicron* and tryptophanase-negative *Bacteroides caccae* and then mice were fed with a fructooligosaccharide diet for 2-weeks. At the end of the study, it was reported that fructooligosaccharide supplementation provides gut microbiota modulation in favor of *Bacteroides caccae* over the *Bacteroides thetaiotaomicron* strain. In consequence of this change of gut microbiota composition, it was observed that urine IS levels significantly decreased [34]. Fructooligosaccharides belong to fructan-type prebiotics like inulin. However, fructooligosaccharide is short-chain, while inulin is long-chain β-fructan. It is suggested that the chain length of the fructan is an important factor regarding its fermentability by which bacterial strain [35]. In the present study, we used oligofructose-enriched inulin, which consists of short and long-chain fructans.

Chronic systemic inflammation is one of the most important reasons for CKD development and progression [36, 37]. Dysbiotic gut microbiota can cause local and systemic inflammation in CKD, through increasing permeability of intestinal mucus [38], increasing the ratio between intestinal T helper type 17 and regulatory T cells [39], and causing translocation of active lipopolysaccharides and gut bacterial products into the systemic circulation [36, 40]. Previous studies have shown that a high level of GDUT can lead to increased pro-inflammatory cytokines release through the production of reactive oxygen species and activation of the nuclear factor-κB pathway [41–43]. In the present study, we examined circulating levels of proinflammatory cytokine IL-6. Our findings indicated that serum IL-6 levels were markedly increased in the CKD group relative to the CTL group. Also, supplementation of oligofructose-enriched inulin led to reduced serum IL-6 levels in CKD rats. It has been shown that prebiotic-resistant starch has a protective effect against inflammation, oxidative stress, renal fibrosis, and CKD progression in other animal experiments [8, 9].

Oxidative stress plays an important role in the progression of the disease. Gene expression and metabolomics data have demonstrated that inflammatory and prooxidant gene expressions were upregulated as well as anti-oxidant system gene expressions were downregulated in patients with CKD [44]. Chen et al. [45] reported that advanced CKD leads to down-regulation of antioxidative stress proteins including GPx, Cu/Zn SOD, catalase in kidney tissue of rats. These findings concur well with the decreased expression of SOD in the CKD group compared with the CTL group observed in our study. GPx expression in the CKD group was lower than that in the CTL group however the difference was not statistically significant (Fig 2). The results of the present study showed that expression of SOD and GPx were significantly upregulated by oligofructose-enriched inulin treatment in CKD rat kidneys (Fig 3). Our results are consistent with previous findings by Vaziri et al. [9] which demonstrated that dietary resistant starch ameliorated oxidative stress via improvement in the expression of antioxidant enzymes, including SOD, GPx, and catalase in kidney tissues of CKD mice. Renal SOD and GPx activity can be expected to be slightly higher in the CTL-Pre group in comparison to the CTL group, as the potential antioxidant effect of prebiotic addition. In the present study, there was no significant difference in SOD and GPx activity between the CTL and CTL-Pre groups (Fig 2). It is speculated that the lack of significant effect of prebiotic supplementation on renal SOD and GPx activity in the CTL-Pre group could be attributed to the absence of oxidative stress activation in undamaged kidney tissues of healthy rats [46]. Although oxidative stress markers such as malondialdehyde and total oxidant status (TOS) were not evaluated in this study, renal SOD activity was significantly lower in CKD rats with adenine-induced kidney damage compared with the healthy rats. Previous studies have indicated that adenine administration significantly increased the oxidative stress markers and reduced the antioxidant enzymes in renal tissue of CKD rats [47, 48]. As mentioned above, the similarity of renal SOD and GPx activity between CTL and CTL-Pre groups was interpreted as these enzyme systems are not rapidly depleted in the absence of renal damage and related oxidative stress.

Uremia has been associated with colonic epithelial barrier dysfunction in an in vitro study of intestinal epithelial cells [49]. Protein-bound uremic toxins, in particular their free forms, accumulate in the intestinal mucosa and can exert toxic effects on colonic barrier integrity [36]. Especially the detrimental effect of PCS on human colonic epithelium has been shown [50]. A marked reduction of colonic tight junction proteins (claudin-1, occludin, and zonula occludens-1) levels has been reported in CKD rats [51]. Our findings did not indicate any significant differences between CKD and CTL rats in terms of occludin or claudin-1 expression in the colon tissues. Although the difference is not statistically different, CKD rats supplemented prebiotic with oligofructose-enriched inulin showed an increasing trend in colonic tight junction protein expression (Fig 4). Whereas Hung et al. [13] found that fermentable dietary fibers, unmodified guar gum, and partially hydrolyzed guar gum have a significant effect on increased expression of colonic tight junction proteins in CKD mice.

Our study has some limitations. First, gut microbiota was not analyzed, so we could not investigate differences in gut microbiota composition among groups. Therefore, microbial species associated with the generation of PCS or IS and which microbial species increased by oligofructose-enriched inulin could not be identified. Second, we only examined free serum concentrations and did not total serum concentrations of the uremic toxins, as well as urine and cecal contents. Another limitation is that we only assessed antioxidant enzyme activity in kidney tissues and did not evaluate oxidative stress parameters. We believe that assessment of oxidative stress and antioxidative enzyme activities together would improve the understanding of injury mechanisms in adenine-induced CKD. Finally, tight junction protein expression in colonic tissues was assessed only by immunohistochemical analysis. It is important to show occludin and claudin-1 expression by immunoblotting, a more informative and sensitive method, alongside immunohistochemical analysis.

In conclusion, consumption of oligofructose-enriched inulin reduced serum urea and free serum concentrations of PCS, ameliorated renal injury, and enhanced the antioxidant enzyme activity in renal tissue of CKD rats. Further clinical studies are needed to evaluate the short- and long-term effects of oligofructose enriched inulin supplementation in patients with CKD.

## Supporting information

S1 Fig Macroscopic view of the kidney of CTL, CTL-Pre, CKD, and CKD-Pre rats. (DOCX)

S1 Table Dataset for physiological and biochemical parameters. (DOCX)

S2 Table Dataset for semi-quantitative scoring of renal histopathological examinations. The degree of nephropathy changes was scored from 0 to 4, (grade 0: no injury, grade 1: <25%, grade 2 = 25–50%, grade 3: 50–75%, grade 4: >75%.
(DOCX)

S3 Table Dataset for quantitative analysis of SOD and GPx positively stained area (%). (DOCX)

S4 Table Dataset for immunoperoxidase expression of occludin and claudin-1 in the ascending and descending colon. Immunohistochemical staining in all groups was scored semi-quantitatively from 0 to 4.
(DOCX)
