# Supplementary figures and images for: Effects of prebiotic oligofructose-enriched inulin on gut-derived uremic toxins and disease progression in rats with adenine-induced chronic kidney disease
Source: PLoS One. 2021 Oct 6;16(10):e0258145. doi: 10.1371/journal.pone.0258145 (PMC8494360; doi:10.1371/journal.pone.0258145)

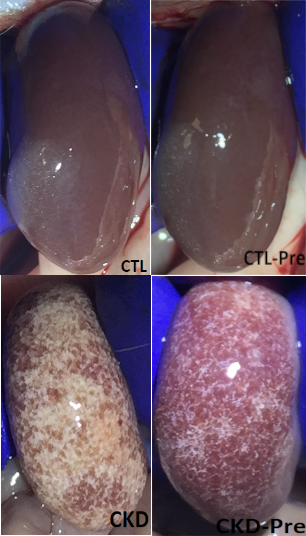

Supplement: S1 Fig — (DOCX) [file pone.0258145.s001.docx]
