# Supplementary material for: Effects of prebiotic oligofructose-enriched inulin on gut-derived uremic toxins and disease progression in rats with adenine-induced chronic kidney disease
Source: PLoS One. 2021 Oct 6;16(10):e0258145. doi: 10.1371/journal.pone.0258145 (PMC8494360; doi:10.1371/journal.pone.0258145)
Supplement: S1 Table — (DOCX) [file pone.0258145.s002.docx]

| CTL (n=6) | CTL-Pre (n=6) | CKD (n=8) | CKD-Pre (n=8) |
| --- | --- | --- | --- |
| 438.0 | 452.0 | 352.0 | 417.5 |
| 581.0 | 462.0 | 382.0 | 433.0 |
| 452.0 | 496.0 | 441.0 | 423.5 |
| 513.0 | 482.0 | 286.0 | 364.0 |
| 525.0 | 492.0 | 427.0 | 466.0 |
| 522.0 | 477.0 | 392.0 | 414.5 |
|  |  | 401.3 | 388.6 |
|  |  | 322.0 | 463.5 |

Body weight (g)

Food intake g/day

| CTL (n=6) | CTL-Pre (n=6) | CKD (n=8) | CKD-Pre (n=8) |
| --- | --- | --- | --- |
| 30.9 | 29.4 | 20.0 | 29.3 |
| 36 | 27.7 | 25.2 | 29.8 |
| 29.7 | 35.9 | 30.2 | 29.6 |
| 34.5 | 29.9 | 18.6 | 26.4 |
| 32.6 | 29.8 | 30.9 | 30.0 |
| 34.3 | 28.9 | 28.2 | 26.7 |
|  |  | 28.5 | 22.7 |
|  |  | 22.6 | 28.5 |

| CTL (n=6) | CTL-Pre (n=6) | CKD (n=8) | CKD-Pre (n=8) |
| --- | --- | --- | --- |
| 35.1 | 46.9 | 164.8 | 62.8 |
| 37.9 | 36.5 | 94.6 | 59.9 |
| 46.1 | 42.2 | 89.7 | 45.7 |
| 42.7 | 39.5 | 190.1 | 71.6 |
| 44.8 | 33.1 | 63.3 | 72.6 |
| 45.0 | 36.0 | 153.8 | 100.1 |
|  |  | 163.2 | 97.7 |
|  |  | 100.0 | 74.9 |

Serum urea (mg/dl)

| CTL (n=6) | CTL-Pre (n=6) | CKD (n=8) | CKD-Pre (n=8) |
| --- | --- | --- | --- |
| 0.91 | 0.41 | 1.15 | 0.89 |
| 0.9 | 0.4 | 1.11 | 0.57 |
| 0.48 | 0.45 | 0.72 | 0.91 |
| 0.61 | 0.43 | 1.82 | 0.86 |
| 0.5 | 0.47 | 0.6 | 0.93 |
| 0.38 | 0.51 | 1.65 | 0.83 |
|  |  | 1.96 | 1.03 |
|  |  | 1.25 | 0.6 |

Serum creatinine (mg/dl)

Serum PCS (ng/ml)

| CTL (n=6) | CTL-Pre (n=6) | CKD (n=8) | CKD-Pre (n=8) |
| --- | --- | --- | --- |
| 682.69 | 362.97 | 2478.99 | 566.3 |
| 1275.14 | 531.63 | 1350.1 | 954.23 |
| 1213.95 | 244.88 | 1560.42 | 1104.79 |
| 585.56 | 244.6 | 2617.66 | 217.54 |
| 831.64 | 447.86 | 966.09 | 488.85 |
| 820.34 | 275.18 | 992.46 | 237.99 |
|  |  | 2863.11 | 321.66 |
|  |  | 1339.62 | 681.81 |

| CTL (n=6) | CTL-Pre (n=6) | CKD (n=8) | CKD-Pre (n=8) |
| --- | --- | --- | --- |
| 83.56 | 84.12 | 171.45 | 118.03 |
| 92.6 | 105.22 | 127.96 | 101.35 |
| 93.26 | 94.25 | 99.26 | 101.74 |
| 83.65 | 94.06 | 135.09 | 122.05 |
| 91.96 | 88.46 | 103.74 | 100.7 |
| 90.2 | 102.54 | 162.75 | 87.97 |
|  |  | 161.22 | 91.36 |
|  |  | 111.23 | 94.65 |

Serum IS (µg/ml)

Serum IL-6 (pg/ml)

Serum IL-10 (pg/ml)

| CTL (n=6) | CTL-Pre (n=6) | CKD (n=8) | CKD-Pre (n=8) |
| --- | --- | --- | --- |
| 108.66 | 90.65 | 281.82 | 34.87 |
| 32.09 | 16.68 | 218.46 | 80.15 |
| 14.93 | 16.83 | 141.58 | 144.09 |
| 67.17 | 17.12 | 301.18 | 75.07 |
| 40.34 | 15.51 | 149.12 | 108.65 |
| 14.93 | 198.79 | 272.12 | 17.85 |
|  |  | 85.49 | 26.49 |
|  |  | 196.28 | 16.98 |

| CTL (n=6) | CTL-Pre (n=6) | CKD (n=8) | CKD-Pre (n=8) |
| --- | --- | --- | --- |
| 41.38 | 23.6 | 29.03 | 27.24 |
| 37.89 | 22.99 | 46.56 | 27.84 |
| 60.68 | 29.63 | 45.41 | 35.55 |
| 115.87 | 40.21 | 253.68 | 78.34 |
| 67.37 | 79.99 | 81.64 | 97.83 |
| 32.01 | 65.15 | 78.9 | 97.29 |
|  |  | 199.18 | 67.37 |
|  |  | 95.67 | 22.37 |
