# Supplementary material for: Effects of prebiotic oligofructose-enriched inulin on gut-derived uremic toxins and disease progression in rats with adenine-induced chronic kidney disease
Source: PLoS One. 2021 Oct 6;16(10):e0258145. doi: 10.1371/journal.pone.0258145 (PMC8494360; doi:10.1371/journal.pone.0258145)
Supplement: S2 Table — The degree of nephropathy changes was scored from 0 to 4, (grade 0: no injury, grade 1: <25%, grade 2 = 25–50%, grade 3: 50–75%, grade 4: >75%. (DOCX) [file pone.0258145.s003.docx]

|  | Crystalline deposition | Tubular damage | Glomerular damage | Glomerular inflammation | Interstitial fibrosis | **Total injury score** |
| --- | --- | --- | --- | --- | --- | --- |
| CTL (n=6) | 0 | 1 | 1 | 0 | 0 | 2 |
|  | 0 | 1 | 0 | 0 | 0 | 1 |
|  | 0 | 1 | 0 | 0 | 0 | 1 |
|  | 0 | 0 | 0 | 0 | 0 | 0 |
|  | 0 | 0 | 0 | 0 | 0 | 0 |
|  | 0 | 1 | 1 | 0 | 0 | 2 |
| CTL-Pre (n=6) | 0 | 1 | 0 | 0 | 0 | 1 |
|  | 0 | 1 | 0 | 0 | 0 | 1 |
|  | 0 | 0 | 0 | 0 | 0 | 0 |
|  | 0 | 1 | 0 | 0 | 0 | 1 |
|  | 0 | 0 | 0 | 0 | 0 | 0 |
|  | 0 | 0 | 0 | 0 | 1 | 1 |
| CKD (n=8) | 2 | 4 | 3 | 3 | 4 | 16 |
|  | 4 | 4 | 4 | 4 | 4 | 20 |
|  | 1 | 2 | 3 | 3 | 3 | 12 |
|  | 3 | 4 | 4 | 4 | 4 | 19 |
|  | 4 | 3 | 4 | 3 | 4 | 18 |
|  | 4 | 4 | 4 | 4 | 4 | 20 |
|  | 3 | 4 | 4 | 4 | 4 | 19 |
|  | 4 | 4 | 4 | 4 | 3 | 19 |
| CKD-Pre (n=8) | 2 | 1 | 1 | 2 | 1 | 7 |
|  | 1 | 2 | 1 | 2 | 2 | 8 |
|  | 0 | 1 | 1 | 2 | 1 | 5 |
|  | 1 | 2 | 2 | 2 | 3 | 10 |
|  | 1 | 3 | 2 | 4 | 3 | 13 |
|  | 2 | 3 | 2 | 3 | 3 | 13 |
|  | 1 | 2 | 2 | 2 | 3 | 10 |
|  | 0 | 1 | 1 | 1 | 1 | 4 |
