# Supplementary material for: Effects of prebiotic oligofructose-enriched inulin on gut-derived uremic toxins and disease progression in rats with adenine-induced chronic kidney disease
Source: PLoS One. 2021 Oct 6;16(10):e0258145. doi: 10.1371/journal.pone.0258145 (PMC8494360; doi:10.1371/journal.pone.0258145)
Supplement: S3 Table — (DOCX) [file pone.0258145.s004.docx]

|  |  | Mean | S.D | SEM | p value |
| --- | --- | --- | --- | --- | --- |
| SOD | CTL (n=6) | 13.9783 | 5.09201 | 1.20020 | <0.001 |
|  | CTL-Pre (n=6) | 17.3911 | 6.39934 | 1.50834 |  |
|  | CKD (n=8) | 6.4094 | 3.16823 | 0.79206 |  |
|  | CKD-Pre (n=8) | 10.6524 | 4.15503 | 0.77157 |  |
| GPx | CTL (n=6) | 13.9400 | 4.91293 | 1.74 | 0.01 |
|  | CTL-Pre (n=6) | 16.3808 | 4.19576 | 1.48343 |  |
|  | CKD (n=8) | 9.7261 | 1.69286 | 0.59852 |  |
|  | CKD-Pre (n=8) | 14.7733 | 3.56957 | 1.18986 |  |
