# Supplementary material for: Effects of prebiotic oligofructose-enriched inulin on gut-derived uremic toxins and disease progression in rats with adenine-induced chronic kidney disease
Source: PLoS One. 2021 Oct 6;16(10):e0258145. doi: 10.1371/journal.pone.0258145 (PMC8494360; doi:10.1371/journal.pone.0258145)
Supplement: S4 Table — Immunohistochemical staining in all groups was scored semi-quantitatively from 0 to 4. (DOCX) [file pone.0258145.s005.docx]

|  |  | Mean | S.D | SEM | p value |
| --- | --- | --- | --- | --- | --- |
| Ascending colon occludin | CTL (n=6) | 1.6250 | 0.51755 | 0.18298 | 0.04 |
|  | CTL-Pre (n=6) | 2.0000 | 0.81650 | 0.30861 |  |
|  | CKD (n=8) | 1.1250 | 0.35355 | 0.12500 |  |
|  | CKD-Pre (n=8) | 1.5556 | 0.52705 | 0.17568 |  |
| Descending colon occludin | CTL (n=6) | 1.3750 | 0.51755 | 0.18298 | 0.881 |
|  | CTL-Pre (n=6) | 1.3750 | 0.51755 | 0.18298 |  |
|  | CKD (n=8) | 1.2857 | 0.48795 | 0.18443 |  |
|  | CKD-Pre (n=8) | 1.5 | 0.53452 | 0.18898 |  |
| Ascending colon claudin-1 | CTL (n=6) | 1.8571 | 0.37796 | 0.14286 | 0.001 |
|  | CTL-Pre (n=6) | 3.4286 | 0.78680 | 0.29738 |  |
|  | CKD (n=8) | 1.6250 | 0.91613 | 0.32390 |  |
|  | CKD-Pre (n=8) | 2.5556 | 0.88192 | 0.29397 |  |
| Descending colon claudin-1 | CTL (n=6) | 1.6250 | 0.51755 | 0.18298 | 0.156 |
|  | CTL-Pre (n=6) | 2.3750 | 0.74402 | 0.26305 |  |
|  | CKD (n=8) | 1.7500 | 0.88641 | 0.31339 |  |
|  | CKD-Pre (n=8) | 2.1250 | 0.64087 | 0.22658 |  |
